# Enabling Access to Medical and Health Education in Rwanda Using Mobile Technology: Needs Assessment for the Development of Mobile Medical Educator Apps

**DOI:** 10.2196/mededu.5336

**Published:** 2016-06-01

**Authors:** Jean Christophe Rusatira, Brian Tomaszewski, Vincent Dusabejambo, Vincent Ndayiragije, Snedden Gonsalves, Aishwarya Sawant, Angeline Mumararungu, George Gasana, Etienne Amendezo, Anne Haake, Leon Mutesa

**Affiliations:** ^1^ College of Medicine and Health Sciences, University of Rwanda Kigali Rwanda; ^2^ Rochester Institute of Technology Rochester, NY United States

**Keywords:** mobile medical education, technology, user-centered design

## Abstract

**Background:**

Lack of access to health and medical education resources for doctors in the developing world is a serious global health problem. In Rwanda, with a population of 11 million, there is only one medical school, hence a shortage in well-trained medical staff. The growth of interactive health technologies has played a role in the improvement of health care in developed countries and has offered alternative ways to offer continuous medical education while improving patient's care. However, low and middle-income countries (LMIC) like Rwanda have struggled to implement medical education technologies adapted to local settings in medical practice and continuing education. Developing a user-centered mobile computing approach for medical and health education programs has potential to bring continuous medical education to doctors in rural and urban areas of Rwanda and influence patient care outcomes.

**Objective:**

The aim of this study is to determine user requirements, currently available resources, and perspectives for potential medical education technologies in Rwanda.

**Methods:**

Information baseline and needs assessments data collection were conducted in all 44 district hospitals (DHs) throughout Rwanda. The research team collected qualitative data through interviews with 16 general practitioners working across Rwanda and 97 self-administered online questionnaires for rural areas. Data were collected and analyzed to address two key questions: (1) what are the currently available tools for the use of mobile-based technology for medical education in Rwanda, and (2) what are user's requirements for the creation of a mobile medical education technology in Rwanda?

**Results:**

General practitioners from different hospitals highlighted that none of the available technologies avail local resources such as the Ministry of Health (MOH) clinical treatment guidelines. Considering the number of patients that doctors see in Rwanda, an average of 32 patients per day, there is need for a locally adapted mobile education app that utilizes specific Rwandan medical education resources. Based on our results, we propose a mobile medical education app that could provide many benefits such as rapid decision making with lower error rates, increasing the quality of data management and accessibility, and improving practice efficiency and knowledge. In areas where Internet access is limited, the proposed mobile medical education app would need to run on a mobile device without Internet access.

**Conclusions:**

A user-centered design approach was adopted, starting with a needs assessment with representative end users, which provided recommendations for the development of a mobile medical education app specific to Rwanda. Specific app features were identified through the needs assessment and it was evident that there will be future benefits to ongoing incorporation of user-centered design methods to better inform the software development and improve its usability. Results of the user-centered design reported here can inform other medical education technology developments in LMIC to ensure that technologies developed are usable by all stakeholders.

## Introduction

Over the last 20 years, continuous professional development (CPD) and continuing medical education (CME) have developed significantly. Greater emphasis has been put on the needs of patients and respective needs at different points of care. CME has now been influenced by the expectations of patients towards health care providers rather than individual needs of doctors. This has been an efficient CME model and has strengthened the knowledge of doctors in particular practice areas [1]. In Rwanda, with a population of over 11 million [2], there is only one medical school from which around 100 new doctors graduate every year. Further, medical practitioners have little access to CME and CPD after their studies. However, given the rapid evolution of new research and developments in all areas of medical care and CME, it is imperative that professionals continue to update their knowledge and skills regularly [3].

In many countries, there is interest in improving the health system through CPD, CME, and Web-based education systems for health care professionals and patients [4]. One of the efficient ways that have been used, but not currently in Rwanda, is Web-based systems. The evolution of Internet-based tools for sharing digital information for public access has increased the availability of online resources with the capacity to continuously share updates and CME tools [5]. A good number of health care professionals are using the Internet and mobile phones to locate medical education information and knowledge [4]. It has been suggested that an important range of use is directly related to questions that arise from patient care.

A variety of interactive health technologies are being used to deliver asynchronous and synchronous forms of Web-based CME [4,6]. Various models for Web-based CME learning have also been reported, furthering education and guiding diagnosis based on clinical symptoms [5]. The role of the Internet as a source of information for health care professionals is very significant whether accessed on computers, tablets, or mobile phones in Rwanda. However, one factor that has contributed to this development is the actual increasing information needs of doctors and patients.

The dearth of access to recent updates and evidences from different medical studies around the world is a challenge for the majority of health care professionals who are expected to maintain their knowledge on the most recent advances in medicine. Due to the limited number of specialized health care facilities in Rwanda, a large number of patients consult primary health care facilities. Patients who consult such facilities potentially leave with unanswered questions; while doctors are also left with one question for every 15 patients approximately [7]. For doctors working in low resource settings more questions will also arise not only because of the types of patients but also the diversity of diseases that are encountered [8]. Timely access to relevant information and updates is key for providing timely answers to these questions and offer the best evidence-based medical care [9].

The Rwandan health care system has evolved into patient-centered care and evidence-based medicine practice while improving the quality of medical education. Examples of the latter include a revision of the curriculum of the school of medicine, initiation of post-graduate specialty training in the major medical specialties and sub-specialties, and providing the legal mandate for physician licensing and specialty certification to the Rwanda Medical and Dental Council [3]. Since 1995, some CME events have been available through the annual conferences of the Faculty of Medicine and the Rwanda Medical Association, together with occasional conferences sponsored by professional medical associations [3]. However, many physicians have not been able to participate in these conferences. Furthermore, there have not been any structured and ongoing educational activities based on identified needs of Rwandan health care professionals. Given the rapid evolution and new research in all areas of medical care, physicians, nurses, and other health professionals must continue to update their knowledge and skills on a regular basis to keep up with the benefits of new health technologies as well as applying evidence-based medicine [3].

The evolution of the Internet as a worldwide connectivity tool has been key to the adoption of information and communication technology (ICT) globally. Building on this opportunity, people have designed platforms that allow for the sharing of information and interactions between professionals. While ICT has been explored much more in business, it is now being used more than ever in the domain of medicine (curative and preventive). With the increase of computer literacy and medical knowledge available on the Internet in our communities, health care professionals must be well prepared to cope with changing patient behaviors and knowledge [10]. Information technology has provided medical students and professionals with more user-friendly access to a significant quantity of information. However, computing skill levels have also impeded the use or adoption of ICT and computer-based tools. Worldwide, medical educators now use technology more than ever to deliver learning resources leading to a better understanding on the role of technology in CME and its impact on the point of care [10]. Unfortunately, these ICT developments have not reached low and middle-income countries (LMIC), like Rwanda, despite the ever-ending increase of patients’ demands and medical practice that need to remain at the global standard. A good number of technology-based tools are widely available but they are not adapted to the local settings and users have not been consulted for the development of tools [11].

Within this background, we argue that a user-centered development (UCD) approach is needed to ensure that mobile medical education developments are relevant and useful. This method has been proven to increase the usability of computer systems and interactive health technologies (IHTs) in general [12,13]. A variety of tools are designed to address specific challenges and in order to reach their goals they have to fit with the expectations of the users while being comfortably usable [14]. Efficacy, satisfaction, and effectiveness are key factors of the usability of technology-based tools and these factors have to be fulfilled for tools to be tailored to meet the educational needs of users, especially in the health fields [15]. Potential user requirements should be the major focus for mobile software interface design, especially in medical education. Required interface features should dominate the design of the rest of the system [16]. However, not only are there no descriptions on whether or how the apps used for medical education in Rwanda involve users in their developments but also there are no reports on their efficacy in improving their knowledge and practices. It is rare to find reports describing how different IHT tools were developed, especially the methods that were used to involve users in the early stages to design and test their usability [11].

### Context

Rwanda is facing a problem of insufficient medical education institutions as well as limited technology-based training tools to provide CME to health care professionals. There is only one medical school with insufficiently qualified medical educators. The doctor to population ratio was 1 to 15,428 in 2012 with an annual population growth of 2.9% [17]. In addition to the geographical distribution of health care facilities and insufficient resources for CME, the previously mentioned factors play a big role in poor health care quality. There is great need in CPD by which health professionals are kept updated to meet the needs of patients, the health care service, and their own professional development. We believe that this can only be achieved as a result of efficient interactive technology-based CME and access to needed information and updates. However, in order to develop a useful CME tool, it is important to determine the needs of health care providers, and the nature, characteristics, and content of the current Web-based tool in the continuous acquisition of new knowledge, skills, and attitudes for graduated and future health care professionals [18]. In this regard, a team of professionals and students from the University of Rwanda and the Rochester Institute of Technology (RIT) undertook a user needs assessment study with the expected end product being a well-functioning user-centered technology-based tool, facilitating CME and better care practices in Rwanda. This paper reports results of the user needs assessment research that is part of the larger study.

### User Centered Design Framework

One of the efficient models for developing apps is UCD. This approach involves user-centered activities throughout the whole process of developing the software [19]. UCD is complex and involves a variety of methods. There is a broad spectrum of applying the UCD but the most important factor is involving users in one way or another. On the one hand, some types of UCD consult potential users about their requirements from the early stages of the design process, mostly during user needs gathering and usability testing. On the other hand, there are UCD methods in which users provide a series of feedbacks about the technology under development, hence playing a significant role throughout the whole design process [19]. Studies suggest that the role of users in the design and development of a new technology should not be underestimated because their involvement will improve the technology’s quality due to a more accurate assessment of user requirements, recommendations, and potential factors to a higher level of user acceptance [20]. Involving users has also been found to substantially reduce development time because usability problems are identified and resolved before these tools are launched [21]. For example, applying UCD to the development of IHTs for patients has been found to improve functionality and usability, therefore increasing the likelihood of promoting the intended health behaviors and health outcomes [11].

Three principles have been recommended for efficient UCD: (1) focusing on users and expected tasks, (2) empirical measurement, and (3) iterative design. With respect to focusing on users and expected tasks, it is first crucial that designers have a good understanding of who the users will be. This understanding can only be achieved by directly studying user behavioral, anthropometric, and attitudinal characteristics and adapting these characteristics to the respective local settings taking into considerations the work that needs to be accomplished. For the empirical measurement, it is key that early in the development process, intended users should be involved through usability testing and prototypes trials to carry out real work, and their performance and reactions should be observed, recorded, and analyzed. As part of the iterative design, problems found during the usability testing have to be fixed through redesigning [14].

This paper describes the findings from a baseline study and needs assessment for the development of a user-centered mobile medical education tool. The tool is focused on the Rwandan medical education system with a particular focus on satisfying the needs of medical doctors practicing in public hospitals from across Rwanda.

### Applying User Centered Design

Throughout the development of the mobile medical educator in Rwanda app, key principles and stages for UCD were applied (Table 1). Participants of the baseline study and the needs assessment were chosen to match the expected end users; medical doctors working in different urban and rural hospitals throughout the country. This model has been proven to be efficient in developing other IHTs [13].

Table1. Principles and stages of the user-centered design and their transfer to the mobile medical educator app.

**Table:** 

Number	Principle	Rwanda mobile medical educator app
P1	Understand the user, task, and environment requirements	Choose appropriate metrics: baseline study and needs assessment
P2	Encourage early and active involvement of users	Interaction between users and developers to develop first version of prototype
P3	Be driven and refined by user-centered evaluation	Valid evaluation metrics
P4	Include iteration of design solutions	Continuous interaction between developers and end users in their home environment leading to several prototypes
P5	Address the whole user experience	Evaluation metrics that covers all aspects “usability” (ie, effectiveness, efficiency, satisfaction)
P6	Encourage a multi-disciplinary design	Identify need and potential impact
S1	Understand and specify the context of use	Identify need and potential impact through the baseline study
S2	Specify the user requirements for medical education, knowledge sharing and decision making support	Questionnaires and interviews for needs assessment
S3	Produce design solutions to meet user requirements	Prototypes available for testing of usability
S4	Evaluate the designs against requirements	Evaluation metrics (effectiveness, efficiency, satisfaction)

## Methods

A team from the School of Medicine of the College of Medicine and Health Sciences at the University of Rwanda, Medfoster, comprised of senior consultants from different fields of medicine, doctors, and one resident conducted the baseline study through self administered questionnaires and interviews where possible. The needs assessment was conducted in collaboration with a team from the RIT, a university that is well distinguished in technology, consisting of two faculty and two students. The baseline study and needs assessment lasted for two and three months, respectively.

### Site Selection and Sampling

For reasons of efficiency in collecting users’ requirements, we chose to work in all 44 district hospitals (DHs) of Rwanda. Data collection for the baseline was completed over a 2-month period by the Medfoster team. Two doctors were randomly selected from the list of doctors working in the hospital and they were requested to fill out the online baseline questionnaire through a phone call. All conversations were confidential. Respondents were informed that the conversation will be part of a research project aimed at developing a mobile technology for interactive continuous medical education, knowledge sharing, and decision making support, and not an evaluation or assessment. Participants gave informed consent and agreed to fill out the questionnaire by phone call or online by checking the “Yes” box. For the interviews, the conversations were conducted in the fluent language of the respondent. The baseline questionnaire to doctors dealt with their daily activities and experiences, their training, and work experience; special training programs, availability of medical resources and updates, available forms of medical information, their recommendations and perspective on availability of medical information, and updates potentially via Web- and mobile-based technology. The needs assessment was conducted through guided open-ended interview question to 16 medical doctors from randomly selected hospitals in all five different regions of the country.

### Preparation Workshop of the Study Team

To orient the research team members who conducted the interviews, the research director conducted a 1-day training workshop in Kigali. The training included a presentation on the research design and background including (1) a review of the objectives, research questions, and methodology of the baseline study; (2) a discussion of the principles of UCD; and (3) the questionnaire to be used for the baseline study. This questionnaire was discussed and finalized in this workshop. Although all of the participants already had substantial experience in collecting qualitative and quantitative data, they benefited from the discussion of the UCD approach that eventually was very useful and oriented them throughout all the stages of the study.

### Organization of Fieldwork

Four weeks before the beginning of fieldwork, the School of Medicine and Pharmacy College of Medicine and Health Sciences Ethics Committee approved the project. Letters were sent to the districts' health offices requesting their support of the project. Randomly selected doctors to participate in the baseline study and the needs assessment were contacted by phone one week before the administration of the questionnaire or the interview. At the same time the leaders of concerned health facilities were contacted for confirmation.

### Data Collection

During the 1-day training with the research director, the questionnaire to be used for the baseline study was piloted with three medical doctors working in one Muhima DH located in the capital city, one of the 44 DHs that were covered by the study. After this pilot, the research team met in order to assess the efficiency of the tools and any need of amendments. After the training with the study director, the research assistant led the baseline study data collection online, and other trained Medfoster members conducted interviews in hospitals where the questionnaires could not be filled in online. The focus of the data collection was to gain an understanding of the currently available medical resources and opportunities that are not well explored through the mobile medical educator app. The research team collected baseline information from 97 medical doctors of whom 83 were self-administered online questionnaires and 14 were through interviews. The research team analyzed the Web-based baseline survey to find recurring thematic patterns for further investigation of the themes in face-to-face interviews during the needs assessment interviews.

For the needs assessment, in order to motivate the medical doctors to talk about their experiences, training, and their perspectives about a mobile-based tool in an open-ended way, the research assistants from Rwanda and RIT used a 2-page conversational outline in academic languages (English and French) that showed general introductory questions to be asked in the same way to everyone, as well as a list of related topics to be covered in any way appropriate. After, the audio-recorded interviews collected data were transcribed and the research assistant developed summaries for each respondent. After reviewing the summaries and transcripts, the research team developed a draft outline to guide the writing of the report.

## Results

### Baseline Survey Findings

**Figure 1:**
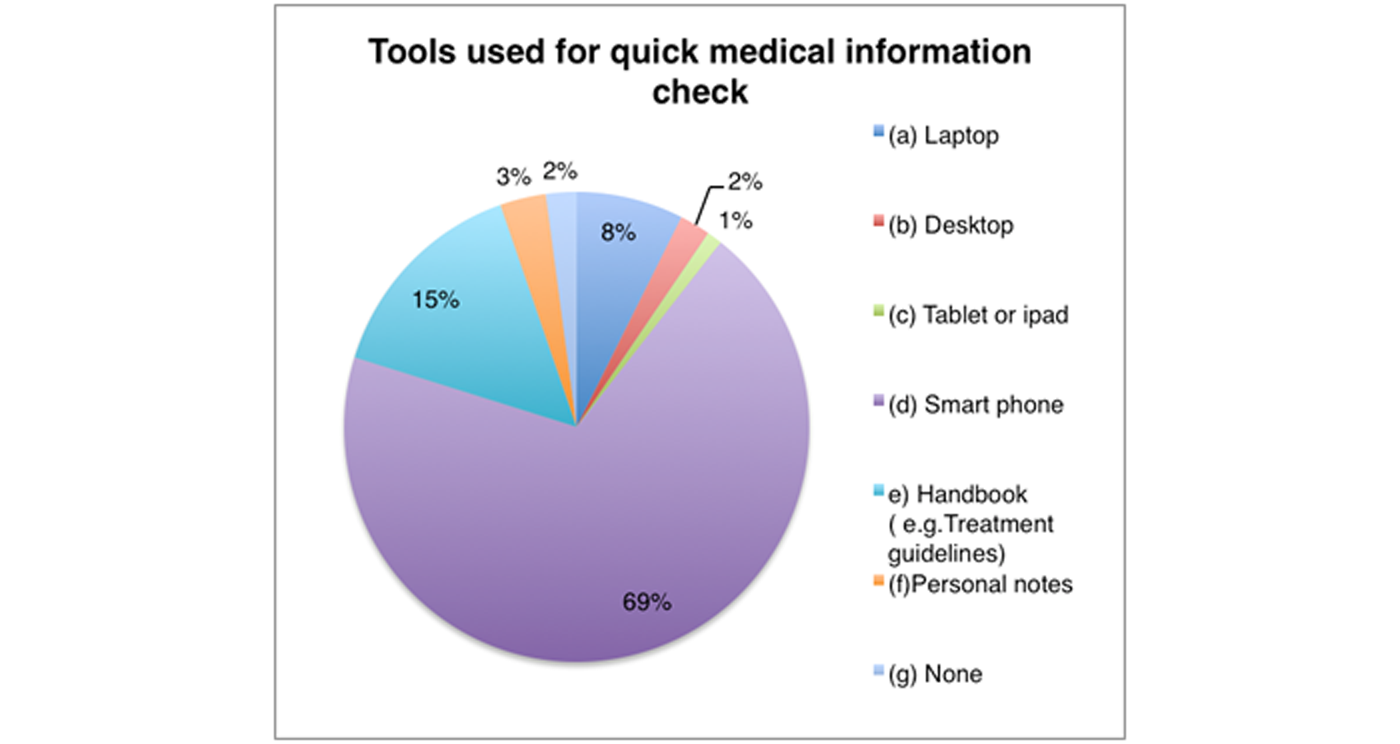
Doctors’ means of accessing the Internet in hospitals.

**Figure 2:**
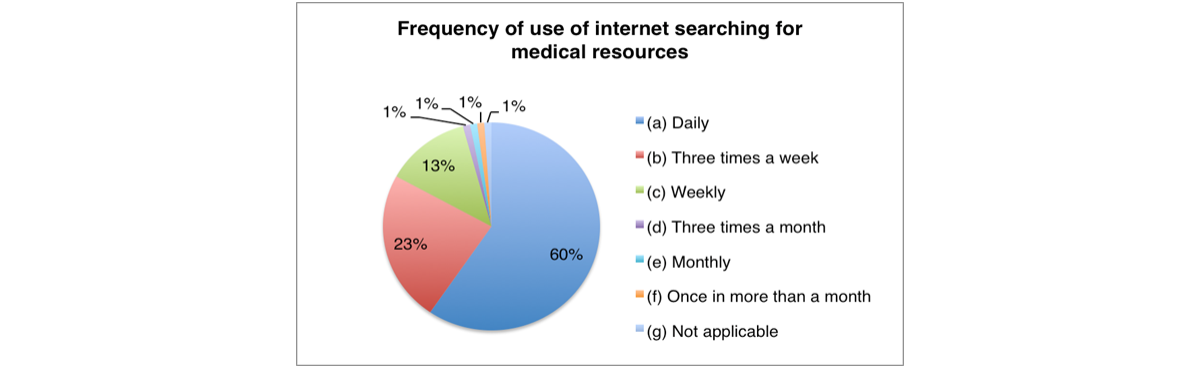
Internet use for medical resources.

A total of 581 medical doctors work in 44 different DHs throughout the country according to the records of the Rwanda Medical and Dental Council (RMDC). The majority of them (66.1%, 384/581) are young with ages that vary between 20 to 30 years, working experience between 2 and 5 years, and predominantly male (81.1%, 471/581). All doctors who filled in the online form reported daily Internet use but not always for medical education purposes. The tools used to check for medical information vary but mobile phones (69%, 67/97) and handbooks (15%, 15/97) are used the most, while tablets are used the least (1%, 1/97) (Figure 1). In addition, accessibility to hospital computers and books is also a big challenge considering that the majority of hospitals do not have computer labs or libraries. The most widely used information resources are the Ministry of Health (MOH) clinical guidelines books but the majority (57%, 55/97) of study respondents indicated they are not satisfied with these resources. Nevertheless, as alternative to library challenges, most doctors (60%, 58/97) reported daily use of the Internet specifically searching for medical resources (Figure 2). Social media is also very frequently used for medical education purposes with 69% (67/97), 18% (17/97), and 10% (10/97) using YouTube, Facebook, and Twitter, respectively.

**Figure 3:**
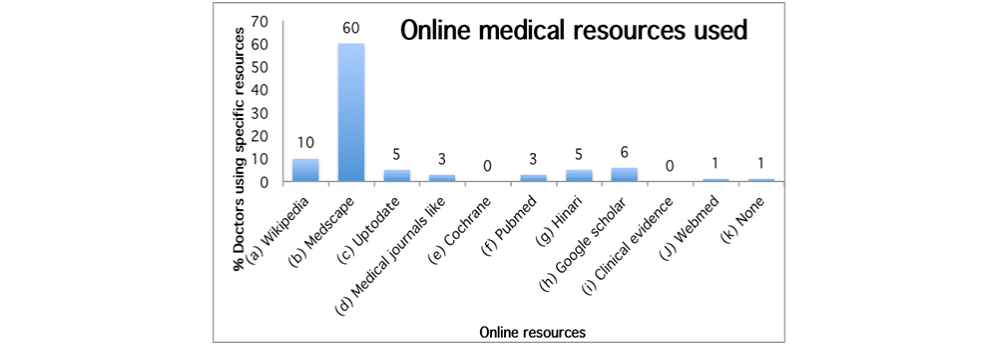
Medical resources used from the Internet.

**Figure 4:**
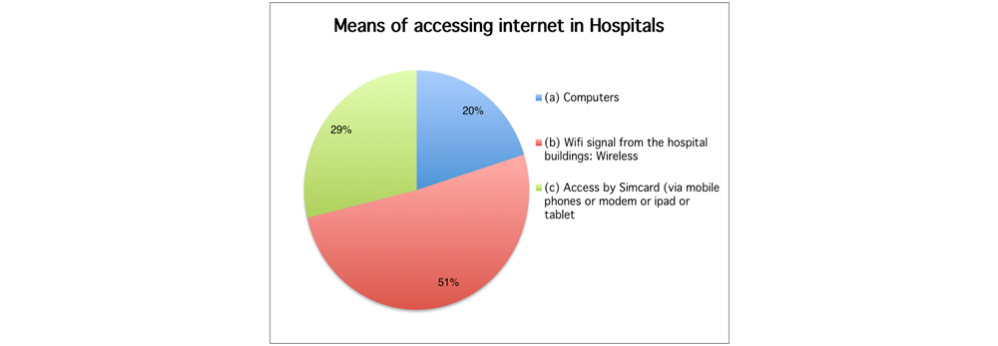
Means of accessing the Internet.

The average number of patients seen per doctor was found to be 32 patients per day and the mostly used online medical information tools are Medscape, Wikipedia, Google Scholar, Hinari, and UpToDate (Figure 3). Half of respondents (51%, 49/97) had access to wireless Internet (WiFi) in their hospitals and 29% (28/97) could only access online resources by subscriber identification module (SIM)-card powered devices, namely mobile phones, modems, and tablets (Figure 4).

### Needs Assessment Interview Findings

In order to collect in depth information on user requirements for potential technology, qualitative information was collected from 16 doctors and interviews were conducted in the languages preferred by respondents. The most common languages used in the Rwandan health care provision system are English and French, and each language was preferred by 8 of the 16 doctors. Interviewed doctors have been working for a span of 2 months to 6 years in a particular hospital and depending on how long they have been there, they were able to give us a bird's eye view of the technological changes, medical education tools, knowledge sharing opportunities, and decision-making support available to them.

### Medical Education Tools

**Figure 5:**
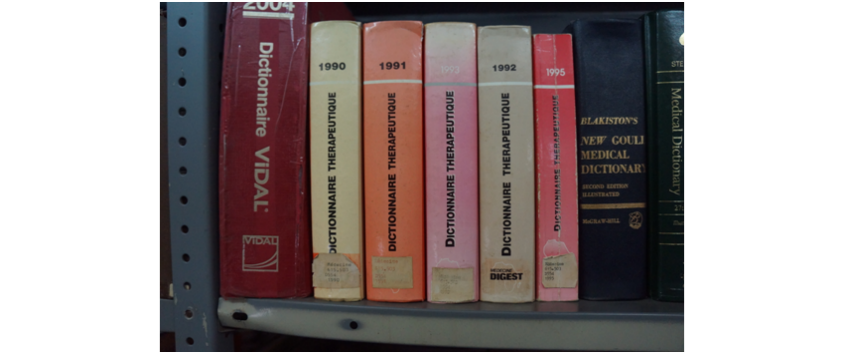
Examples of outdated medical books in a Rwandan hospital library.

Transcription analysis revealed that in one of the hospitals there is a small library, which primarily holds only donated, old, and outdated versions of national treatment guidelines and books (Figure 5). This means that the doctors have limited resources if they want to check out health guidelines or recent treatment options, as the following quote from one 39 years old general practitioner indicates:

We have a small library with old books. They are not enough.

It was revealed that the Internet is not widely available in all hospitals considering the fact that many hospitals do not have stable wireless connections; 10 doctors reported that they have wireless connectivity at their hospitals whereas 6 doctors reported that they don’t have Internet access which makes them vulnerable to less frequent updating of medical techniques and resources. In those cases, hospitals have modem connections, which are slow and can cause bottlenecks in accessing information on-demand. Some doctors who heavily rely on the Internet for their medical information use a SIM card on their mobile phones to access information on the go.

The majority of the users (81%, 13/16) primarily prefer soft copies to hard copies of books for accessing medical information because it is fairly easy: “I prefer online and soft copies in general because it’s easy to consult many books at the same time and they are carry in one compound.”

Of the doctors older than 50 years of age, 3% (3/97) responded to hardly use the Internet and are unaware of the availability of online resources for medical education purposes. Nevertheless, the number of online resources used (Figure 3) clearly demonstrates that doctors seek access to medical information irrespective of their exposure level. The needs assessment interviews revealed that when interacting with patients, Medscape (preferred by 4 doctors) and UpToDate (preferred by 3 doctors) are the most preferred apps for quick information access. Medscape has an edge over UpToDate partly because it can be accessed offline with limited features whereas UpToDate requires Internet connectivity, which makes it undesirable when on the field mentoring patients.

From the baseline study, the following users requirements germane to development of a mobile medical education tool emerged: (1) means to have quick reference for evidence-based consultations, based on symptoms, keywords searches, or direct content access, (2) English and French language support, (3) access and study the most recent medicine practice and knowledge, (4) prefer an organized way of searching and locating information, similar to Medscape, and (6) forum for information exchange and sharing.

### Knowledge Sharing Opportunities

Most of the users heavily use Android mobile phones and laptop computers as a means of staying connected, and accessing and sharing information. Tablet and iPad usage is now becoming common among doctors and is the preferred gadget or tool for accessing and sharing information on the go (50%, 8/16 doctors). Accessing new knowledge and guidelines is a challenge for practicing doctors. No official track or platform by which new evidence-based practices are communicated was found. Further, respondents were not aware of how frequent the MOH guidelines are updated. Update notifications are mostly sent via group emails among doctors and sometimes through word of mouth through colleagues. The World Health Organization (WHO) Health Guidelines are updated every two years and these updates are communicated through the MOH together with updates through the MOH guidelines.

The following users needs regarding knowledge sharing emerged from the interviews: (1) means to share knowledge about unknown diseases or issues as well as treatments applied and techniques results, (2) platform that connects all doctors in Rwanda to discuss difficult and complicated cases for figuring out the best treatment option for the patient, (3) platform that provides content both with Internet access (Full access) and without Internet Access. (Limited access), (4) forum for information exchange and sharing, and (5) prefer update notifications through emails.

### Decision-Making Support

General practitioners reported that they see around 28 to 36 patients on a daily basis and the inpatient stay is around 5 days in a week, on average. Almost all doctors (94%, 15/16) use an Android phone and are currently constantly connected to other doctors through WhatsApp for effective knowledge sharing, gaining support from colleagues, and discussions about unusual or complicated cases. Some other media platforms used in these regards are email and Facebook: “We have an e-group for sharing information.”

In addition, the following users needs regarding decision-making support emerged from the interviews with doctors: (1) means to post questions and answer other professionals’ questions, (2) forum should potentially support attachment of images (ie, x-rays, echoes, scans) for consultation purposes, (3) list of available doctors nearby to connect with for emergency cases discussions, and (4) rate if a given content or support was helpful.

## Discussion

### Medical Education Functionality

The baseline study and the needs assessment clearly showed that doctors are eager to easily access medical information irrespective of their available facilities and exposure level. Despite great efforts by the government of Rwanda in availing computers and Internet in different hospitals, the need is still far from being satisfactorily addressed.

While the hospitals do not hold libraries, the doctors often consult, most of the time on their mobile phones, online resources to answer their questions and to keep themselves updated with modern medical techniques. It was found that the main available and accessed tools are Medscape, Wikipedia, Google Scholar, Hinari, and UpToDate. However, these mostly used apps do not conform to the MOH clinical guidelines, and they are accessed separately. For doctors in remote locations, computers with Internet access are not always available; therefore, no access to Internet resources is even possible. The end users need of tools that may ease their access to the national MOH guidelines and updated medical resources was elucidated, with the fact that none of the used apps avail these guidelines and availing these resources on phones or tablets would be ideal for health care professionals: “I would love to know everything through my cell phone.”

### Knowledge Sharing Functionality

Mobile phones with app capabilities are widely used and Rwandan doctors have been able to access a number of Internet-based tools. Numerous apps are now available to assist health care providers with many of their daily important tasks, such as information, health record, communications and consulting, reference and information gathering, patient management and monitoring, clinical decision-making, and medical education and training [22]. There is a great opportunity of advancing technology in the medical field in Rwanda, considering that the majority of Rwandan doctors are mainly young and of whom 60% reported daily use of the Internet to check for medical information.

### Decision-Making Support Functionality

The number of patients per doctor per day is still very high in Rwanda. This also entails the number of unanswered questions they get from those patients. One option for consultants mainly based in cities to offer decision making support especially to doctors in remote areas would be through a technology-based platform that may be able to overcome the challenge of Internet that those doctors face. As the following quote indicates:

I wish there was a platform which bring together all medical doctors to share medical information like complicated and interesting cases to learn from each other. This would be a good opportunity for knowledge sharing from generations to generations.

Mobile technology-based diagnosis and management have been found most relevant to health care providers in developing countries where mobile phones potentially allow clinical support and evidence-based guidance to be delivered to health care professionals working remotely and in circumstances where senior health care professional support or other infrastructure is lacking [23]. This technology will be useful and will not only improve the knowledge it will also prevent outdated practices, reduce medical decision errors, and promote more evidence-based practices as the following quote indicates: “We don’t have a specific way or channel through which we can get information about updates (Local) in medicine.”

### User Classes and Characteristics

A key finding from the baseline study and needs assessment interviews is the identification of user classes and characteristics that can inform the development of a mobile medical education tool. For example, the tool will need a consistent solution that can impact pre-determined user classes differently. Furthermore, elicitation and analysis were made from the previously discussed baseline study and interviews in order to identify how different user classes have interests in using the system and system features. The results of the analysis are presented in Textbox 1.

**User classes and characteristics.**

Interests and frequently used functionalities

1. Doctors

  a. Major interests

    i. View and share knowledge on recent developments in Medicine and interesting findings from patient cases and other studies

    ii. Ask for information and opinions

    iii. Consults external resources and platforms pointed by the system

  b. Frequently used functionalities

    i. Discuss cases, view resources, check guidelines

    ii. Communicate with other doctors

    iii. Update recommended resources

2. Administrators

  a. Major interests

    i. Monitor app

    ii. Ensure updated content, resolve user issues, and maintain privileges

  b. Frequently used functionalities

    i. Update user privileges

    ii. Update content (eg, MOH guidelines)

### Key User-Required Features for the Mobile Medical Educator Technology

Both the baseline and needs assessment revealed the key features of a much needed Web-based app that can be accessed through the most popular tools used by doctors, mobile phones, computers, and tablets (Textbox 1). The proposed platform will be a central hub for aggregating content from valuable medical practice information to medical education resources that spread the newest and effective techniques starting from the local MOH guidelines.

A list of essential features was identified and the mentioned features are presented in Textbox 2.

**List of essential features.**

Feature

- Edit discussion threads
- Search discussion threads with keywords or medical specialty
- Delete discussion threads
- Create patient case discussion record
- Edit patient case record
- Search patient case by keyword or medical specialty
- Delete patient case record
- Provide external resources and platforms with medical content by topics and medical specialties
- Comment discussion threads or patient records

### Use Case Model

**Figure 6:**
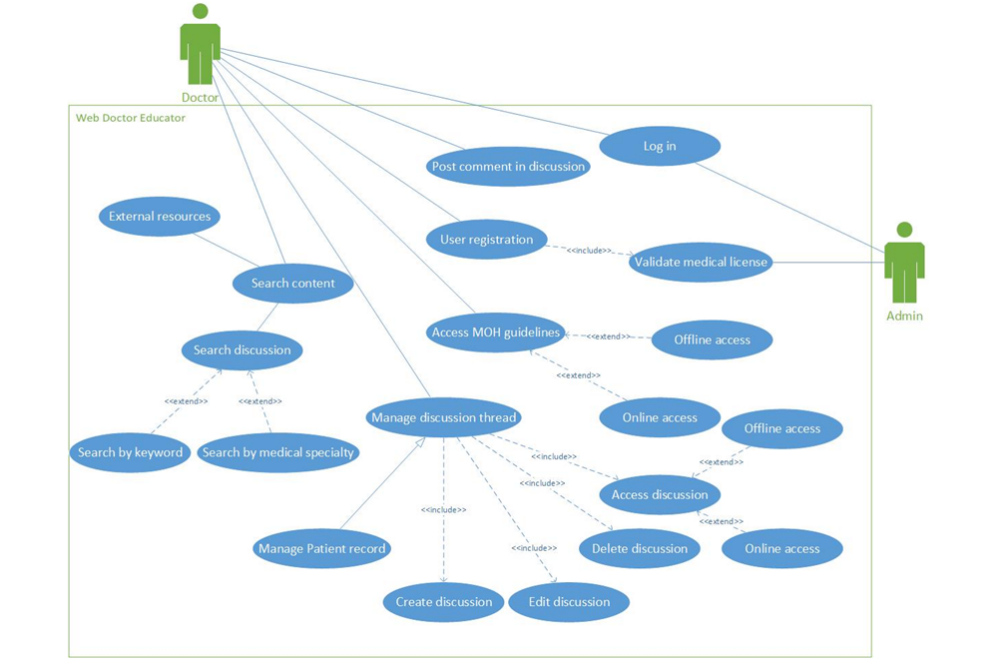
Use case diagram for the mobile medical educator application for Rwandan medical doctors.

In order to better analyze the users’ interaction with the system features a use case modeling was performed. Our model organizes the features according to the dimensions of the envisioned mobile medical education app. The modeling is presented in Figure 6 and Table 2. Details on UC1 to UC16 can be found in Multimedia Appendix 1.

**Table 2:** Detailed use cases.

ID	Name	Description
UC1	User registration	This use case describes how to sign up for the app
UC2	User login	This use case describes how to sign in for the app
UC3	Validate medical license	This use case describes how the app will validate doctors credentials with MOH
UC4	Access MOH guideline documentation	This use case describes how a doctor consults the MOH guidelines in the app
UC5	Create discussion thread	This use case describes how a doctor discusses a general topic with fellow doctors
UC6	Search discussion thread	This use case describes how a user searches for a discussion thread
UC7	Access patient case studies	This use case describes how a user accesses a patient discussion thread
UC8	Archive discussions and case studies for offline access	This use case describes how the doctor makes discussion available for offline use
UC9	Update experienced doctor privileges	This use case describes how the app administrator updates the users with experienced doctor privileges
UC10	Edit discussion thread	This describes how to edit existing discussion thread
UC11	Delete discussion thread	This use case describes how a user will delete a discussion thread
UC12	Edit a patient case record	This use case describes how a user can edit a patient case record
UC13	Search a patient case record	This use case describes how a user can search for a patient case record
UC14	Post a comment to a discussion thread	This use case describes how the doctor adds a new post/comment to discussion thread
UC15	Delete a patient case record	This use case describes how the system deletes a patient’s case discussion record
UC16	Create a patient case record	This use case describes how a doctor creates a patient record discussion

### Future Work

The next step in our research is to build and test a mobile medical education tool based on the findings and insights reported in this paper. Unlike previous medical resources and platforms used, the technology we will develop will serve as a viable one-stop shop for daily use by Rwandan doctors, grouping a variety of quality and reliable medical information resources. This platform has potential to reform medical education and practice from a traditional lecture and discussion system to a more learner-centered and evidence-based type of education with many benefits at different points of care. In addition, the system will be specifically targeted to the Rwandan doctors and their need of information and continuous education, as well as being available on the most popular and accessible platforms in the country. It was vital for the study team to assess currently available tools, gaps, and opportunities for improvement. Key user recommended features were identified and deepened through user modeling, and will be the basis for ongoing design and implementation of this technology. Furthermore, considering the number of patients that doctors see in Rwanda, in the long-term this technology has promise to provide many benefits such as enabling more rapid decisions with a lower error rate, increasing the quality of data management and accessibility, and improving practice efficiency and knowledge, especially in areas where access to the Internet is compromised. However, it is vital that throughout the process, principles and stages of UCD are well followed so as to have final software that fulfills the needs of the users.

### Conclusions

Through the conducted baseline study and needs assessment, it was determined that almost all Rwandan doctors engage in daily Internet use. The tools used to access the Internet vary but mobile phones and laptops are mostly used, with mobile phones being the most popular. It was also found that very few of the hospitals have libraries and the most used information resources are the MOH guidelines books. However, many survey respondents indicated that they are not satisfied with these resources. The majority of the hospitals do not have computer labs or libraries. Despite this challenge, surveyed doctors reported that they use MOH guidelines books and mobile phones for quick information checks for evidence-based practice. A great need for mobile-based technology for medical education exists for Rwandan doctors and health care professionals throughout the country who need quick and easy access to medical information to answer their daily patient-related questions, and who also need access to updates and information about the most effective and modern medical techniques. An online platform for medical education in Rwanda should aggregate a wide variety of the most used medical resources by Rwandan doctors and health care professionals. It could also direct to external resources of most recent techniques and knowledge in the medical field, accessible via the most popular platforms and devices in Rwanda starting from the MOH clinical guidelines.

## Appendix

Multimedia Appendix 1 Details on UC1 to UC16.

## Abbreviations

CME: continuing medical education
CPD: continuous professional development
DH: district hospital
ICT: information and communication technology
LMIC: low and middle-income countries
MOH: Ministry of Health
RIT: Rochester Institute of Technology
SIM: subscriber identification module
UCD: user-centered development
