# Supplementary material for: Enabling Access to Medical and Health Education in Rwanda Using Mobile Technology: Needs Assessment for the Development of Mobile Medical Educator Apps
Source: JMIR Med Educ. 2016 Jun 1;2(1):e7. doi: 10.2196/mededu.5336 (PMC5041362; doi:10.2196/mededu.5336)
Supplement: Multimedia Appendix 1 [file mededu_v2i1e7_app1.pdf]

Annex A. Use Case Models

1.1 Use Case 1: Register as a New User

Use Case ID:	UC1		
Use Case Name:	User registration		
Created By:	Richard Kalimba	Last Updated By:	Iago da Silva
Date Created:	03/01/2015	Date Last Updated:	03/14/2015

Actors:	Doctor (Regular user)
Description:	This use case describes how to sign up for the application
Trigger:	New application use,
Preconditions:	1. Doctor has medical credentials
Normal Flow:	1. Application displays the register/sign in page with user's name and password fields<br>2. The doctor selects to register<br>3. The application display the profile information page and form<br>4. The doctor enters the information: user-name, first name, last name, email used for medical council correspondence, identification number, repeated password(strong pattern) , hospital, location, phone<br>5. the doctor saves information<br>6. the application system validates the information completeness, and against MOH and Medical Council data<br>7. the application sends link to verify email, or code to phone<br>8. the application displays code page while suggesting to also verify email<br>9. the user verifies email, or enters code in text field<br>10. the application displays the home screen
Alternative Flows:	6. the application fails to validate information and displays error reason<br>7. the application goes back to edit profile screen
Exceptions:	
Includes:	
Priority:	High
Frequency of Use:	
Business Rules:	
Special Requirements:	
Assumptions:	
Notes and Issues:	

1.2 Use Case 2:User Login

Use Case ID:	UC2		
Use Case Name:	User Login		
Created By:	Waleed Zogaan	Last Updated By:	
Date Created:	03/14/2015	Date Last Updated:	

Actors:	Doctor (Regular user)
Description:	This use case describes how to sign in for the application
Trigger:	Application use
Preconditions:	**1.** Doctor already registered.<br>**2.** System is running<br>**3.** Internet connection is working
Postconditions:	**1.** Doctor able to login.<br>**2.** Application is showing home screen.
Normal Flow:	**1.** Application displays the register/sign in page with user's name and password fields<br>**2.** The doctor selects to login<br>**3.** The application display the login page and form<br>**4.** The doctor enters the user-name and Password.<br>**5.** The doctor click Login button.<br>**6.** The application system validates the information.<br>**7.** The application displays the home screen
Alternative Flows:	A.F 1:<br>6. The application fails to validate password and display "Incorrect password, Please enter your password" message.<br>7. The application goes back to Register/Login page.<br>A.F 2:<br>6. The application fails to validate user-name and display

	"User-name was not found" message.<br><br>7. The application goes back to Register/Login page.
Exceptions:	
Includes:	
Priority:	High
Frequency of Use:	Daily
Business Rules:	
Special Requirements:	
Assumptions:	
Notes and Issues:	

1.3 Use Case 3: Validate Medical License.

Use Case ID:	UC3		
Use Case Name:	Validate Medical License		
Created By:	Waleed Zogaan	Last Updated By:	
Date Created:	03/14/2015	Date Last Updated:	

Actors:	Administrator, MOH database, WDE
Description:	This use case describes how the application will validate Doctors credentials with MOH.
Trigger:	New user registration
Preconditions:	1. Doctor already registered.<br>2. System is running<br>3. Internet connection is working
Postconditions:	1. Doctor credentials was confirmed.

	2. Application is showing home screen.
Normal Flow:	1. User click 'Submit' button in the New User Form.<br>2. The application connect to MOH database.<br>3. The application Validate the New User information and Identification number with the MOH records.<br>4. New user information are valid.<br>5. The application send confirmation email to the user.<br>6. User is activated.
Alternative Flows:	A.F 1:<br>4. New user information are not valid.<br>5. The application send "Validation Failed" email to the user.<br>6. User is deactivated.
Exceptions:	
Includes:	
Priority:	High
Frequency of Use:	Daily
Business Rules:	
Special Requirements:	
Assumptions:	
Notes and Issues:	

1.4 Use Case 4: Access the MOH Guidelines

Use Case ID:	UC4		
Use Case Name:	Browse the MOH Guidelines		
Created By:	Richard Kalimba	Last Updated By:	Iago da Silva
Date Created:	03/01/2015	Date Last Updated:	03/14/2015

Actors:	Doctor (Regular User)
Description:	This use case describes how a doctor consults the MOH guidelines in the application
Trigger:	Need to check guidelines
Preconditions:	1. The doctor is logged in.
Postconditions:	1. The MOH guidelines is displayed.
Normal Flow:	1. From the home screen of the application the choice of access to MOH Guidelines displayed<br>2. The user selects the MOH guideline<br>3. The application displays a screen with collapsed hierarchy of sections and chapters of the document<br>4. Based on the subject of interest the doctor expands each part of the hierarchy<br>5. The desired section of pages is reached and displayed by the application<br>6. From a given page the doctor can navigate and scroll through content, or collapse section again
Alternative Flows:	
Exceptions:	
Includes:	

Priority:	High
Frequency of Use:	Many times daily
Business Rules:	
Special Requirements:	
Assumptions:	
Notes and Issues:	

1.5 Use Case 5: Create discussion thread

Use Case ID:	UC5		
Use Case Name:	Discuss a general medical topic		
Created By:	Richard Kalimba	Last Updated By:	Richard Kalimba
Date Created:	03/01/2015	Date Last Updated:	03/03/2015

Actors:	Doctor
Description:	This use case describes how a doctor discusses a general topic with fellow doctors
Trigger:	Any new idea or question from doctor about the medical practice
Preconditions:	**1.** The doctor has a live Internet connection.<br>**2.** The doctor is logged in.
Postconditions:	**1.** Relevant doctors are notified of new discussion
Normal Flow:	**1.** The application shows the home screen with a selection that includes option to open Discussions<br>**2.** The application goes to Discussions Board<br>**3.** The doctor selects from discussions board to Discuss a general topic<br>**4.** The application displays screen with Topic Categories: Internal Medicine, Surgery, Pediatrics, Obstetrics & Gynecology.<br>**5.** The doctor selects a topic category<br>**6.** The application shows a text field to post the discussion with option for media attachment. (Attach Media Use Case)<br>**7.** The doctor shares case details by editing the contents of text field and optionally attaches media<br>**8.** The doctor selects the share option<br>**9.** The application opens the discussion board for the new topic (View Discussion Board Use Case)
Alternative Flows:	
Exceptions:	Exceeds post length limit

Includes:	Attach Media
Priority:	
Frequency of Use:	Many times daily
Business Rules:	
Special Requirements:	
Assumptions:	
Notes and Issues:	

1.6 Use Case 6: Search discussion

Use Case ID:	UC6		
Use Case Name:	Search discussion threads with keywords or medical specialty.		
Created By:	Iago da Silva	Last Updated By:	Iago da Silva
Date Created:	03/14/2015	Date Last Updated:	03/14/2015

Actors:	Doctor (Regular User)
Description:	This use case describes how a user searches for a discussion thread.
Trigger:	Need to learn more about medical practices, or discussion conclusions.
Preconditions:	1. Doctor is logged in the system
Postconditions:	1. A set of discussions is displayed as the search result
Normal Flow:	1. The application shows the home screen with a search box on top.<br>2. Doctors type medical specialty or keywords and submit the search<br>3. The application displays the search result.
Alternative Flows:	
Exceptions:	
Includes:	
Priority:	

Frequency of Use:	Many times daily
Business Rules:	
Special Requirements:	
Assumptions:	
Notes and Issues:	

1.7 Use Case 7: Access patient case studies

Use Case ID:	UC7		
Use Case Name:	Access patient case studies.		
Created By:	Richard Kalimba	Last Updated By:	Iago da Silva
Date Created:	03/01/2015	Date Last Updated:	03/14/2015

Actors:	Doctor
Description:	This use case describes how a user access a patient discussion thread.
Trigger:	New discussion notification.<br>Updated discussion notification.<br>Doctor needs to know more information about the treatment of a specific case.
Preconditions:	1. Doctor is logged in.
Postconditions:	1. The selected patient case is displayed with details.
Normal Flow:	1. The application shows the home screen with a patient records options.<br>2. The doctor selects patient records options<br>3. The doctor selects one of the patient records
Alternative Flows:	A.F 1:<br>1. The doctor searches for a patient record<br>2. The doctor selects one of the search result<br>AF 2:<br>1. The doctor receives notification about a patient record.<br>2. The doctor selects the patient record in the notification<br>3. The patient record discussion is displayed with details.
Exceptions:	
Includes:	
Priority:	
Frequency of Use:	
Business Rules:	
Special Requirements:	
Assumptions:	
Notes and Issues:	

1.8 Use Case 8: Archive discussions and case studies for offline access

Use Case ID:	UC8		
Use Case Name:	Archive discussions and case studies for offline access.		
Created By:	Richard Kalimba	Last Updated By:	Iago da Silva
Date Created:	03/01/2015	Date Last Updated:	03/14/2015

Actors:	Doctor
Description:	This user case describes how the doctor makes discussion available for offline use.
Trigger:	The doctor wants to use a discussion offline.
Preconditions:	1. User is logged in the system<br>2. There are available discussion threads
Postconditions:	1. The discussion thread can be visualized offline
Normal Flow:	1. The user executes Use Case Access patient case studies, or Use Case Access discussion thread<br>2. The doctor selects options in the discussion<br>3. The doctor selects make available offline
Alternative Flows:	
Exceptions:	
Includes:	Use Case Access patient case studies<br>Use Case Access discussion thread
Priority:	Medium
Frequency of Use:	Medium
Business Rules:	
Special Requirements:	
Assumptions:	
Notes and Issues:	

1.9 Use Case 9: Update Experienced Doctor Privileges

Use Case ID:	UC9		
Use Case Name:	Update Experienced Doctor Privileges		
Created By:	Richard Kalimba	Last Updated By:	Iago da Silva
Date Created:	03/01/2015	Date Last Updated:	03/14/2015

Actors:	Admin
Description:	This use case describes how the application administrator updates the users with experienced doctor privileges
Trigger:	
Preconditions:	1. the user has administrator privileges
Postconditions:	1.
Normal Flow:	1. The application home screen shows options that include manage users<br>2. the admin selects the option to manage users<br>3. the application displays screen with List of users as panel with two sections, and option to move back and forth users from/to privilege given section (Regular Doctors or

	Experienced Doctors)<br>4. The admin selects user(s) to be moved either way<br>5. The admin selects move option<br>6. The application asks for confirmation of the update to move user in different privilege group<br>7. The admin confirm privileges changes<br>8. the application shows the list of users in respective privilege section
Alternative Flows:	
Exceptions:	
Includes:	
Priority:	
Frequency of Use:	
Business Rules:	
Special Requirements:	
Assumptions:	
Notes and Issues:	

1.10 Use Case 10: Edit Discussion Thread.

Use Case ID:	UC10		
Use Case Name:	Edit Discussion Thread		
Created By:	Waleed Zogaan	Last Updated By:	
Date Created:	03/14/2015	Date Last Updated:	

Actors:	Doctors
Description:	This use case describes how to edit existing discussion thread.
Trigger:	The doctor wants to edit a discussion thread.
Preconditions:	1. User has internet connection.<br>2. Application is running.<br>3. User is logged in.
Postconditions:	1. Discussion thread edited successfully.<br>2. Edited discussion thread is posted.
Normal Flow:	1. The application shows the home screen with a selection that includes option to open Discussions<br>2. The doctor navigates to Discussions Board<br>3. The doctor selects discussion thread from discussions board.<br>4. The doctor selects a discussion thread.<br>5. The application shows a text field to edit the discussion with option for media attachment. (Attach Media Use Case)<br>7. The doctor edit thread details by editing the contents of text field and optionally attaches media<br>8. The doctor selects the post option<br>9. The application post edited thread.
Alternative Flows:	A.F 1:<br>8. The doctor selects the cancel option<br>9. The application clear the text fields.

Exceptions:	Exceeds post length limit
Includes:	Attach Media
Priority:	
Frequency of Use:	Many times daily
Business Rules:	
Special Requirements:	
Assumptions:	
Notes and Issues:	

1.11 Use Case 11: Delete discussion thread

Use Case ID:	UC11		
Use Case Name:	Delete discussion thread.		
Created By:	Amol Pantvaidya	Last Updated By:	
Date Created:	03/14/2015	Date Last Updated:	

Actors:	Doctor
Description:	This use case describes how a user will delete a discussion thread.
Trigger:	The owner of the thread wants to delete the thread.
Preconditions:	1. User already registered.<br>2. Application is able to connect to the internet.<br>3. The thread to be deleted exists
Postconditions:	1. The discussion thread is successfully deleted.<br>2. Application return to the discussion board.
Normal Flow:	1. The user navigates to the discussion board from the home page.<br>2. The user selects the discussion thread to delete.<br>3. The selected discussion thread is displayed along with the operations that could be performed on it.<br>4. The user selects the 'Delete' option.<br>5. The application now verifies if the user is the owner of the thread requested to be deleted.<br>6. The application asks for a confirmation to delete.(Yes/No)<br>7. User selects 'Yes'<br>8. Thread is deleted.<br>9. Application returns to the discussion board.
Alternative Flows:	A.F 1:<br>6. The user is not the owner of the requested thread, Application gives feedback to the user denying access to the operation.<br>8. Application returns to the discussion board.<br><br>A.F 2:<br>7. User selects 'No'.<br>8. Application returns to the thread view screen.
Exceptions:	

Includes:	
Priority:	High
Frequency of Use:	Often
Business Rules:	
Special Requirements:	
Assumptions:	
Notes and Issues:	

3.12 Use Case 12: Edit a patient case record

Use Case ID:	UC12		
Use Case Name:	Edit patient case record		
Created By:	Amol Pantvaidya	Last Updated By:	
Date Created:	03/14/2015	Date Last Updated:	

Actors:	Doctor
Description:	This use case describes how a user can edit a patient case record..
Trigger:	The user wants to edit a patient case record..
Preconditions:	1. User is Logged-in<br>2. Application is able to connect to the internet.<br>3. The requested patient case is logged.
Postconditions:	3. The user is able to edit the requested patient case.
Normal Flow:	1. The user navigates to the patient cases section on the home page.<br>2. The user is able to view all the patient cases logged by the user.<br>3. The user selects the case to edit.<br>4. The case details are displayed to the user in read/write mode.<br>5. The user modifies data as required.<br>6. The user clicks on the 'Save' button.<br>7. The changes to the record are saved.
Alternative Flows:	
Exceptions:	
Includes:	
Priority:	High
Frequency of Use:	Often
Business Rules:	
Special Requirements:	
Assumptions:	
Notes and Issues:	

3.13 Use Case 13: Search a patient case record

Use Case ID:	UC13
Use Case Name:	Search a patient case record.

Created By:	Amol Pantvaidya	Last Updated By:	
Date Created:	03/14/2015	Date Last Updated:	

Actors:	Doctor
Description:	This use case describes how a user can search for a patient case record..
Trigger:	The user wants to modify/view a patient case record..
Preconditions:	1. User is Logged-in<br>2. Application is able to connect to the internet
Postconditions:	1. The user is able to view the requested patient case.
Normal Flow:	1. The user navigates to the patient cases section on the home page.<br>2. The user is able to view all the patient cases logged by the user.<br>3. The top right corner of the current screen provides a search bar to search/filter case records. The user types in the case details in the search box.<br>4. The user selects 'Search' button.<br>5. The requested case is displayed to the user.
Alternative Flows:	AF 1:<br>5. If the requested case is not found in the system the user is given a feedback message- "The requested case could not be found".
Exceptions:	
Includes:	
Priority:	High
Frequency of Use:	Often
Business Rules:	
Special Requirements:	
Assumptions:	The user remembers or has access to certain case details and certain keywords that could be used to search a record.
Notes and Issues:	

3.14 Use Case 14: Post a comment to a discussion thread

Use Case ID:	UC14		
Use Case Name:	Post a comment to a discussion thread		
Created By:	Richard Kalimba	Last Updated By:	Richard Kalimba
Date Created:	03/14/2015	Date Last Updated:	03/14/2015

Actors:	Doctor
Description:	This use case describes how the doctor adds a new post/comment to discussion thread.
Trigger:	The doctor wants to add something on the discussion
Preconditions:	The doctor is signed in the system/application<br>The doctor's device is connected to the Internet, live.
Postconditions:	
Normal Flow:	1. The application shows the home screen with a selection that includes option to open Discussions<br>2. The application goes to Discussions Board

	3. The doctor selects from discussions board to Discuss a Case or a General Topic<br>4. The application displays screen with Topic Categories: Internal Medicine, Surgery, Pediatrics, Obstetrics & Gynecology.<br>5. The doctor selects a topic category<br>6. The application lists all the discussions related to the topic<br>7. The doctor selects to a discussion<br>8. The application displays all the posts/comments to the discussion, with option to add a new comment/post<br>9. The doctor selects to add a new comment<br>10. The application displays an empty text field with option to attach media<br>11. The doctor adds text and optionally attaches media<br>12. The doctor posts the new comment<br>13. The application displays the updated list of posts to the discussion.
Alternative Flows:	
Exceptions:	
Includes:	
Priority:	High
Frequency of Use:	Many times daily
Business Rules:	
Special Requirements:	
Assumptions:	
Notes and Issues:	

3.15 Use Case 15: Delete a patient case record

Use Case ID:	UC15		
Use Case Name:	Delete a patient case record		
Created By:	Richard Kalimba	Last Updated By:	Richard Kalimba
Date Created:	03/14/2015	Date Last Updated:	03/14/2015

Actors:	System
Description:	This use case describes how the system deletes a patient's case discussion record
Trigger:	When it has been a month since the last update to the discussion
Preconditions:	
Postconditions:	The patient case discussions are deleted from system.
Normal Flow:	1. At the end of a week, the system checks for any patient case discussion whose last update is at least a month old.<br>2. The system deletes all patient case discussions whose last update is at least a month old.
Alternative Flows:	
Exceptions:	
Includes:	
Priority:	High
Frequency of Use:	Weekly
Business Rules:	

Special Requirements:	
Assumptions:	After a month without updates, patient case discussions are considered obsolete
Notes and Issues:	

3.16 Use Case 16: Create Patient case discussion record

Use Case ID:	UC16		
Use Case Name:	Create discussion (Patient record)		
Created By:	Richard Kalimba	Last Updated By:	Iago da Silva
Date Created:	03/01/2015	Date Last Updated:	03/14/2015

Actors:	Doctor
Description:	This use case describes how a doctor creates a patient record discussion.
Trigger:	A question when treating a patient
Preconditions:	1. The doctor has a live internet connection<br>2. The doctor is logged in the system
Postconditions:	1. Relevant Doctors receive a notification of a new discussion
Normal Flow:	1. The application shows the home screen with a selection that includes option to open Discussions<br>2. The application goes to Discussions Board<br>3. The doctor selects from discussions board to Discuss a Case<br>4. The application displays screen with Topic Categories: Internal Medicine, Surgery, Pediatrics, Obstetrics & Gynecology.<br>5. The doctor selects a topic category<br>6. The application shows a selectable list of doctors familiar with the topic, and text field to post the discussion with option for media attachment. (Attach Media Use Case)<br>7. The doctor shares case details by editing the contents of text field and optionally attaches media<br>8. The doctor selects the share option<br>9. The application opens the discussion board for the new topic (View Discussion Board Use Case)
Alternative Flows:	
Exceptions:	Exceeds post length limit
Includes:	Attach Media
Priority:	
Frequency of Use:	Many times daily
Business Rules:	
Special Requirements:	
Assumptions:	
Notes and Issues:	
